# When Teratology and Augmented Reality Entwine: A Qualitative Phenomenological Analysis in a Museal Setting

**DOI:** 10.3390/s25123683

**Published:** 2025-06-12

**Authors:** Lucas L. Boer, Frédérique Schol, Colin Christiaans, Jacobus Duits, Thomas Maal, Dylan Henssen

**Affiliations:** 1 Department of Medical Imaging, Section Anatomy and Museum for Anatomy and Pathology, Radboud University Medical Center, 6525 Nijmegen, The Netherlands; 2 Radboudumc 3D Lab, Radboud University Medical Center, 6525 Nijmegen, The Netherlands; 3 Department of Medical Imaging, Section Radiology, Radboud University Medical Center, 6525 Nijmegen, The Netherlands

**Keywords:** anatomy, augmented reality, embryology, museum, teaching, teratology, qualitative

## Abstract

**Highlights:**

**What are the main findings?**

- This is the first qualitative study that shows insights that were encountered when using AR in a teratological collection;
- Both (bio)medical students and general visitors are interested in using AR in an educational and museal setting;
- Teratological exhibitions may enhance public awareness and acceptance of developmental anomalies.

**What is the implication of the main finding?**

- Teratological exhibitions provide a unique opportunity to reflect on both historical and contemporary bioethical issues;
- It was suggested that AR can provide better insights into teratology and create a more interactive way of learning.

**Abstract:**

Background: The Museum for Anatomy and Pathology at the Radboud University (The Netherlands) has created a permanent teratological exhibition, which is enhanced with augmented reality (AR) modalities. This exhibition serves various (post)graduate educational purposes and is open to the general public. However, data on visitors’ views and experiences regarding the teratological collection and AR models are currently lacking. Methods: To address this, a qualitative study was conducted to explore visitors’ opinions and experiences. One-on-one in-depth interviews were conducted using a predefined topic list, with audio recordings transcribed verbatim. Thematic analysis was applied to the twenty-six interview transcripts. Results: The findings indicate that publicly displaying teratological specimens alongside AR modalities is valued and positively received by both (bio)medical students and laypeople alike. AR enhances understanding of dysmorphology and provides a more interactive and engaging learning experience for complex topics. Conclusion: The use of AR within a teratological exposition holds tremendous educational potential and improves public awareness and acceptance of developmental anomalies. Moreover, it provides a unique opportunity to reflect on both historical and contemporary bioethical issues.

## 1. Introduction

Thousands of teratological specimens are maintained and exhibited throughout Europe’s anatomical museums [1,2]. It is in these collections that (bio)medical students and laymen learn about (failing) embryological development and its (peri-natal) outcome. Understanding these gross dysmorphologies is oftentimes performed by sole observation of the teratological specimen itself and distinguished by many as merely difficult because complex dysmorphological processes, which initiated or formed the anomaly in earlier stages, are phenotypically undetectable when observing the external (dys)morphology of preterm specimens or simply unknown enough to most observers [3,4]. Therefore, the field of teaching teratology, which includes many aspects of embryology, holds numerous educational challenges. One of the bigger hurdles for students is their lack of embryological knowledge, which also includes the molecular aspects of human development due to the underrepresentation of these topics in most medical curricula [5]. A large number of complex sequential and interplaying spatial and temporal factors, as well as a multitude of developmental processes during embryological growth, ultimately create and form the anomaly. Ideally, these initiating steps and factors should be recognized and placed into perspective in order to comprehend or conceptualize the congenital anomaly that is observed [5]. As these early morphogenic and embryological concepts remain quite difficult to learn, one possibility to enhance the educational potential of a teratological collection is additional radiological imaging to provide insights into internal (dys)morphology [6,7,8]. This modality is oftentimes the sole addition as it ensures the historical, museal, and anatomical integrity of the specimen as no dissection is required [6]. However, these two-dimensional displays of radiological data are oftentimes difficult to interpret as the (bio)medical student often lacks sufficient ‘radiological overview’ to oversee the sheer volume of (dysmorphological) structures [9]. This creates a possible cognitive overload and an insufficient learning experience. Another approach is the extraction of schematic drawings from these radiological datasets to highlight certain aspects. However, what is lacking in these schematics is their three-dimensional topography and spatial relationship with surrounding structures.

In addition to single images and schematic drawings, so-called ‘walkthrough’ radiological movies can be used to obtain insights into topographical and/or embryological answers. However, this approach still requires radiological and anatomical knowledge to interpret such big datasets. Additionally, the fact that most—if not all—teratological specimens vary significantly from the “normal” anatomical framework makes it even harder to place these modalities in perspective and concur within educational settings. Besides its educational challenges, another issue that is almost intrinsically present when publicly exhibiting teratological specimens is the ethical and moral dilemma most visitors experience [4]. Publicly displaying human remains can cause criticism, controversies, and debate about their educational, ethical, judicial, and/or moral values or grounds [10,11,12]. Oftentimes, these irreplaceable collections house many dysmorphological fetuses, which, for many observers, are considered intangible, repulsive, sensitive, horrendous, and difficult to watch. These feelings and emotions might influence its learning potential. Therefore, these collections are prone to neglect, stigmatization, and matter for provocative disrespectfulness of their onlookers [4]. Unfortunately, the often-made (almost inherently existent) analogy with a cabinet of rarities frequently amplifies the observers’ emotions. Besides these already strong emotions, the fact that many specimens are near or full-term newborns, due to absent prenatal screening during the era in which most specimens were collected, creates awareness of the fact that these museological exhibited objects are children of bequeathed and bereaved parents that became objectified museological elements over time [4]. Except for the emotional-filled reaction of the visitors, wonder, curiosity, and sheer fascination often accompany the mind of the spectator. These feelings of wonder and interest are aspects that any museal exhibition should promote [13]. Besides this, observer-influenced stigmatization, economic downturns, space crunches, and institutional apathy are topical elements that institutionalized medical museums—and therewith teratological collections—have to deal with [14,15]. The abovementioned makes extant teratological collections unique but vulnerable time capsules that show the perceptions, attitudes, and superstitions of past epochs that could be ethically unacceptable from a modern point of view [15,16,17,18]. The way these specimens were collected and publicly displayed, without it being known if consent was given, is oftentimes not accepted in modern-day society. Currently, consent from the parents or any living family member is vital as it shows respect to both the family and their deceased child [17]. On the other hand, these collections are important to treasure and steward because they can house and provide critical insights into anomalies that are so rare that only a handful of specimens have ever been described adequately; the only existing examples are those present in these old medical collections [2,3]. Moreover, these teratological collections have potential for molecular, etiopathogenetic, and developmental studies [4,19]. Finally, they can be exhaustively used to educate teratology and embryology to a broad audience that includes (bio)medical students, residents, and medical specialists from a variety of disciplines, including laymen, albeit the right information, depth, and modality should be used [18,19].

**Figure 1:**
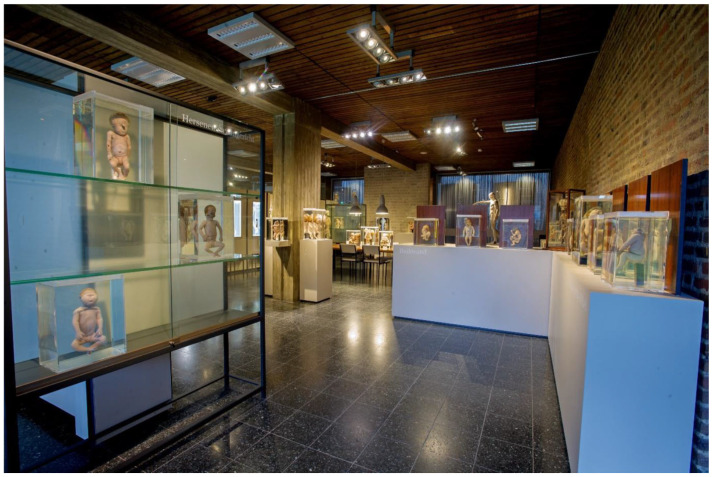
Overview photograph of the teratological collection in the Museum for Anatomy and Pathology of the Radboud University medical center in Nijmegen, The Netherlands. The exhibit, placed in a more secluded area of the museum, displays 36 teratological fetuses with mostly lethal congenital anomalies, specimens of animal teratology, historical books on teratology, physical 3D models, plaster casts, and (digital) information about the exhibited fetuses and teratology in general.

**Figure 2:**
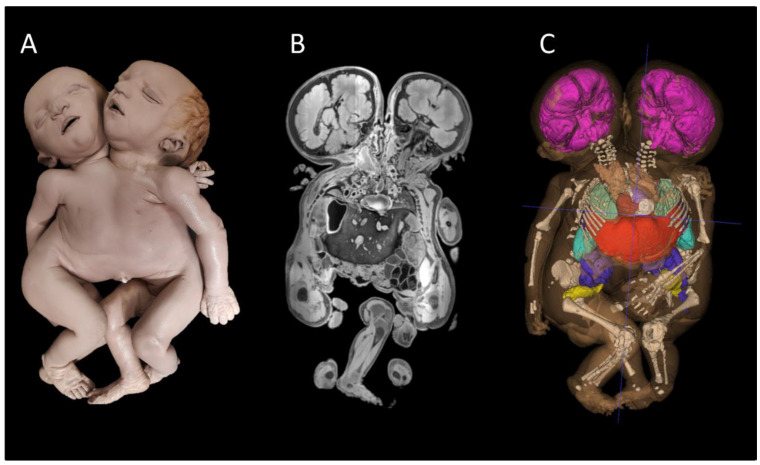
(**A**) External appearance of thoracoileopagus conjoined twins, which is publicly exhibited within the teratological collection. (**B**) Single plane of a T1-weighted MRI of the same specimen showing, i.e., the presence of a single and shared compound liver and heart used in all (post)graduate educational settings. (**C**) Static image of a 3D model that was based on the manual segmentation of the MRI, together with the 3D reconstructed skeleton based on CT data to highlight certain organs (systems) used in all (post)graduate educational settings, which help in the topographical and anatomical overview.

**Figure 3:**
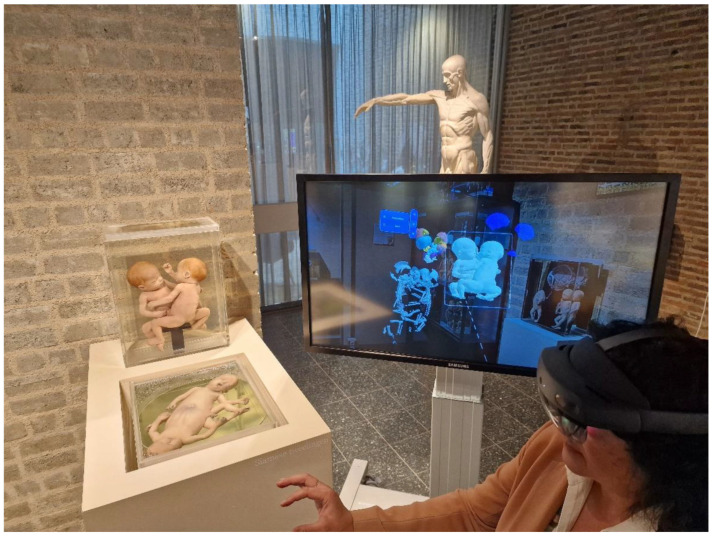
Participant with AR goggles inside the museum looking at an AR model of a caudally united ischiopagus conjoined twins, in which the user interacts with different segmented organs (systems). Specimens could be virtually dissected, and organs could be isolated, inflated, or deflated and studied in depth, after which they could be placed back in the right position. AR classes were linked to a screen so that the instructor (F.S.) could give both technical instructions and anatomical clarity to the user. The real specimen is exhibited on the left.

The museum for Anatomy and Pathology of the Radboud University Medical Center in Nijmegen (The Netherlands) has chosen to create a permanent teratological exhibition that is in use for both graduate and post-graduate educational purposes and publicly accessible and visited by approximately 30,000 visitors a year (Figure 1). The collection was predominantly collected between 1950 and 1980 and houses nearly 75 specimens with a variety of mostly lethal and rare congenital anomalies. In total, 36 of these specimens have been chosen to be permanently placed in the exhibition. MRI and (dynamic 3D reconstructed) CT images of the skeleton, as well as segmented 3D volume renderings of various organ systems of multiple specimens, were created to additionally be used in all educational settings (Figure 2). With this approach, (post)graduates could not only look at the external dysmorphological characteristics of each specimen but also observe their internal characteristics, which, at first thought and experience, help in understanding complex anomalies [8]. However, personal experiences (L.B.) during educational settings within the teratological exhibition led to the fact that the need was present to give more three-dimensional and interactive insight into the internal morphology of a number of specimens to explore the educational potential and boundaries of the teratological collection even more. Therefore, the museum developed and enrolled augmented reality (AR) modalities to accommodate a number of specimens (Figure 3). Various types of AR modalities and techniques have been developed for museal purposes to enhance the potential visitor’s experience [20,21,22,23]. However, as of now, no qualitative data are known to exist on how visitors experience the use of teratological AR modalities within a teratological museal exhibition. For that reason, we conducted a qualitative study in which we interviewed (bio)medical students and general visitors of the Museum for Anatomy and Pathology of the Radboud University Medical Center. The main goal of this qualitative study is to explore the opinions, experiences, and views of (bio)medical students and general museum visitors regarding a teratological collection and the use of AR modalities within such a collection. Part of this objective is to explore the potential role of AR in a museal setting for educational purposes, to engage students and visitors, and to create a more in-depth museum experience. By doing this, the teratological collection is used with its full educational potential and is ready for the next generation, in which innovative and modern initiatives should be clearly present or used.

## 2. Materials and Methods

### 2.1. Ethical Approval

The Regional Review Board for Human Research (METC Oost-Nederland, registration number 3930) assessed the study and judged that ethical approval was not required for this type of study under Dutch national law due to the non-invasive character. Participants were asked for their voluntary participation and written informed consent was obtained at enrollment. All participants signed an informed consent form and were treated in accordance with the principles outlined in the Declaration of Helsinki.

### 2.2. Creation of the AR Models

The AR models used in this study were constructed (C.C./D.D. and T.M.) by using the open-source software package ITK-SNAP, which allows users to create models based on radiological images by means of manual or algorithm-assisted drawing [24,25]. Magnetic resonance imaging (MRI) data were used to manually segment the organ(systems) of interest. Computed tomography (CT) was used to segment the osseous structures. The CT data were linearly transformed with the MRI data of the same specimen using a 12-point affine registration algorithm [26,27]. The manually created and smoothed models were exported to 3D file formats (.obj-files), which can subsequently be used for visualization in AR using an in-house written software package. No additional explanatory texts were included within the models, and they were chosen to purely focus on gross anatomical 3D rendered organs (systems).

### 2.3. Participants and Recruitment

For this qualitative study, participants were eligible for inclusion when they were over 18 years of age and spoke Dutch. Participants consisted of two different groups: (bio)medical students from the Faculty of Medical Sciences at Radboud University Nijmegen, The Netherlands, and general museum visitors. Visitors from this last group consisted of students from non-(bio)medical faculties and other non-student visitors. A purposive sampling strategy was used: possible participants were approached on the (bio)medical campus of the Radboud University or when visitors entered the museum. If visitors were interested in participating, they received more information about the study. Participants were recruited during the period February–April 2024. This approach inherently creates a self-selection bias as individuals with prior interest in AR technology and teratology may, of course, have been more likely to volunteer. To mitigate this, we intentionally did not advertise the study as an “AR experience” but rather as a general interactive museum study. This was with the aim of attracting a broader and more representative visitor demographic. Additionally, we collected demographic data, including participants’ prior experience with AR, which we considered in our analysis to account for potential influence on user responses.

### 2.4. Study Design

To obtain an in-depth understanding of how visitors experience a teratological collection and the use of AR in such a collection, the visitors’ views, opinions, and experiences on these topics were explored using a qualitative research design. Data were collected using semi-structured, one-on-one interviews to obtain different opinions and experiences that can be encountered when visiting a teratological collection and when being exposed to AR modalities in a museal setting. A topic list was constructed (See Supplementary Data) on the basis of the available literature and experiences of two of the researchers with previous qualitative research in medical education (L.B. and D.H.).

The study protocol for participants consisted of four parts: (1) Participants would explore the teratological collection on their own (10 min), (2) Participants were asked to read a short information sheet about the ethical background of the collection (5 min), (3) Participants were provided with a specific etiopathogenetic explanation on three teratological specimens (20 min), and (4) Participants used the Microsoft HoloLens (a head mounted AR device) to view the same three teratological models (20 min). In order to observe the participants’ interaction with the AR modalities, and if necessary, guide participants, the AR device was wirelessly connected to a separate screen. This modus also enabled the researcher to explain the different possibilities of the AR device to the participant and answer any questions they had about the anatomy of the AR models.

After all four steps were completed, face-to-face interviews were conducted in a quiet and neutral environment. Open-ended questions were asked, and participants were encouraged to express their opinions and experiences freely. If needed, clarification was asked to ensure the answers given were understood correctly. A constant comparative method was used in order to include new questions when novel topics arose during the interviews [28]. Images of the teratological specimens and AR modalities were given during the interview in order to refresh their memory on what the participant had just observed during the museal phase of the study.

All interviews were audio-recorded and transcribed verbatim afterward. Additional interviews were conducted until data saturation was reached. After suspected data saturation, two more interviews were conducted for confirmation. These were used to validate the existing thematic framework and to verify that no further relevant themes were emerging.

### 2.5. Data Analysis

The transcribed data from the in-depth interviews were analyzed using a thematic analysis using ATLAS.ti software version 23 for Windows (http://atlasti.com; ATLAS.ti Scientific Software Development GmbH, Berlin, Germany). The first five interviews of both groups were independently coded by two researchers (L.B. and F.S.) to ensure coding reliability. Any discrepancies were discussed until consensus was reached on the codes for the codebook. Analysis took place using an inductive, iterative process and analysis started after the first interview was completed. Codes derived from previous interviews were used as a starting point for subsequent interviews. Several meetings with all three affiliated researchers were held to place and group the codes through the systematic and iterative identification of patterns (axial coding), eventually resulting in the clustering of the codes in order to identify sub-themes and eventually overarching themes. The Consolidated criteria for reporting the qualitative research (COREQ) Checklist were used to guide both the design and analysis of this study [29], as well as the concept of analytic direction and reflexivity, which were contemplated and considered [30].

## 3. Results

### 3.1. Participant Characteristics

In total, 14 (bio)medical students and 12 general visitors were interviewed after their visit to the Museum for Anatomy and Pathology of the Radboud University medical center. Participant characteristics are summarized in Table 1.

### 3.2. Results of the Analysis for (Bio)Medical Students

The (bio)medical students’ experiences and opinions on the teratological collection and the use of AR within this collection were categorized into 11 categories and subsequently sorted into 4 themes (Table 2).

#### 3.2.1. Theme 1: Views on the Teratological Collection

##### Additional Value of the Collection

A visit to the teratological collection is generally described as a positive experience; excitement, interesting, impressive, and extraordinary are terms that are often used to describe the visit. Viewing a teratological collection is also seen as educational and informative because it provides insights into what these rare anomalies look like in real life. They are physical examples showing the visitor all the possibilities for human development. The specimens are an added value to the museum as they can be used to educate both students and general museum visitors.

“*I think these specimens are especially informative because you can see what can go wrong on the outside. Then you realize that it does not only exist in books, but that it also happens in real life*.”

(P3, age 26)

##### Feelings of Unease and Inconvenience

Some students found the teratological specimens scary, strange, and unpleasant to look at. The realness of the specimens is often the component that scares students the most. Another feeling that was often described was sympathy. Students mainly pitied the families of these children.

“*I find it very sad... If you’re the mother of such a child, it must be so strange. You expect to bring a healthy baby into the world, but then you give birth to a deceased child that’s also completely deformed*.”

(P8, age 23)

Furthermore, students found it inconvenient that there was no information on most of the specimens. It was often not clear to them what exactly caused the deformities, but because they possessed anatomical knowledge, they had some idea of what went wrong in the development of the fetus. Information panels or screens with additional information were suggestions on how this information could be conveyed.

##### Ethical Aspects

The most prominent ethical dilemma that presented itself was whether the teratological specimens should be exhibited without parental consent. It is understood that in the past, opinions, norms, and values were different regarding informed consent, but it is troubling that some parents do not know their child is being exhibited to the public. Some students even said that only the specimens with known consent should be available to the general public; specimens without consent should only be available to (bio)-medical students and staff. However, even though no parental consent might have been given in the past, most students regard the specimens as useful to educate both students and general visitors on teratology.

“*I think that not only medical students, but also other groups should know about congenital abnormalities and that things don’t always go well*.”

(P10, age 23)

“*You can say that it is not allowed or wrong or that we shouldn’t do it. But on the other hand, what do you do with them then? I think that now it has already happened, and we can learn valuable information from them*.”

(P7, age 20)

##### Knowledge and Prevention

Teratological specimens are helpful in better understanding the development of congenital abnormalities. They give students a better idea of how these developmental processes are phenotypically expressed, and they clarify the differences and spectrum between congenital anomalies. Exhibiting these specimens creates awareness of the existence, complexity, and severity of these defects. They are also useful in the development of healthcare providers; when they are knowledgeable about different congenital abnormalities, they can recognize and diagnose these defects, provide information to expecting parents, and make better decisions on what is best for the unborn child.

“*Each time when you see it again, it’s strange to realize it does not always go well. We are lucky when it does go well*.”

(P14, age 21)

“*It is something that doctors should learn and understand, because it exists. And because they have to be able to provide information to parents if it happens to them*.”

(P7, age 20)

#### 3.2.2. Theme 2: Positive and Negative Aspects of AR

##### Positive Aspects: Positive Experience

It is fun for students to try new technologies like AR, which enthuses them to learn more about teratology. The use of the AR models was described as unique, cool, amazing, and of added value to both the museum visit and teratology education. Furthermore, students found it very pleasant that they were able to see their own surroundings because this prevented them from feeling cybersickness.

“*It was a lot of fun. I had never done this before, so I found it exceptional that this was even possible and how it’s all made. I think it adds to the experience*.”

(P12, age 22)

##### Positive Aspects: Interactive

AR is an interactive way of learning. You are not just looking at the specimen, you have to be proactive and decide which structure “to grab” and investigate more. Students enjoyed the fact that they could completely disassemble the virtual model. This stimulated them to intrinsically learn and think about the anatomy of the specimens and not just to look at them and move on to the next. This can potentially be useful in both educational and museal contexts. AR models can also be used in a group. You can then share the experience with others and learn from each other. If one person is using the device, other members of the group can watch the screen to which the device is connected, see what structure is indicated, and then discuss the correct answer.

##### Positive Aspects: Insightful

The AR models are useful to observe the inside of the specimens and learn the topography of different organs and systems. It provides insights into developmental processes that are not visible on the outside and helps to visualize the proportions of each structure. These structures can be taken out and viewed from different angles. This is very instructive, especially for a teratology course. Each structure also has its own color, which makes it easier to differentiate them.

“*It’s amazing that you can walk around it, that you can grab it, turn it, that you can take the structures out and enlarge them*.”

(P2, age 27)

##### Negative Aspects: Logistics

When implementing AR in an educational setting or a teratological collection, there are some logistical factors that should be taken into account. The AR devices are expensive and, depending on the amount that is needed, the costs can be high. Software problems are also a worry among students: if the software malfunctions in an educational setting, it needs to be fixed immediately to be able to continue the session properly. Lastly, the use of AR is very time-consuming as the device and its techniques have to be explained before it can be used intuitively.

##### Negative Aspects: Feeling Watched

When working with AR, the people around you can influence your experience. Students sometimes felt awkward when using the AR device because they were manipulating a model others could not see. This can create the feeling that others are watching you. It might also take the focus away from the learning process.

“*There are all these people around me who are not wearing the AR devices, who are just visiting the museum. And I’m there, looking strange with these devices and movements*.”

(P1, age 24)

“*Putting on the glasses and launching the systems is a bit weird. It feels strange because you are in a room, and you have the idea someone is watching you while you’re doing all these things in the air*.”

(P2, age 27)

#### 3.2.3. Theme 3: The Use of AR in a Teratological Collection

##### Impact on Museum Visit

AR modalities can influence the museum experience in multiple ways. Firstly, as AR is a new technology, it could attract people who are interested in AR to visit the museum. Furthermore, AR could create a more positive experience for visitors who find the specimens scary-looking because the AR modalities are not as realistic-looking as the specimens. AR models can be the first step for people to explore, and if those pique their interest, they can then view the specimens in real life. AR also provides the visitor with a better understanding of what happened to the fetuses that are being exhibited. As a result, they are seen as learning objects, which could have a positive impact on their visit.

“*I think this is less shocking for people. So, I think that it’s also more approachable. If people find the specimens really scary, I think this could be more approachable for them*.”

(P9, age 24)

##### AR as an Alternative or Addition to Specimens

AR models can either serve as an alternative or an addition to the teratological specimens. When the AR device is used separately, it could take away the human aspects that are apparent in the specimens. Because the modalities are not as realistic, they could serve as a good alternative to visitors who find the real specimens frightening but still want to learn about teratology. For (bio)medical students, it is important to see the specimens first and understand that these are real children who are being “used” for education. Subsequently, AR can be used to obtain a better understanding of the internal anatomy.

“*I think it’s a good addition to see them next to each other. You have the specimen and that’s what it looks like on the outside. And then you can visualize it even more with the models*.”

(P3, age 26)

As the AR models are described as less realistic, ethical aspects are not as prevalent. However, some students found that because the models are based on the specimens, the ethical dilemmas are similar to those specimens.

“*With the specimens I have the feeling I am looking at someone’s child. With the AR modalities, I am looking at a learning object*.”

(P13, age 25)

#### 3.2.4. Theme 4: Recommendations on Using AR Devices in Museum Tours and Teratology Education

##### User-Friendliness of the AR Device

In general, AR devices are seen as user-friendly devices that can be used by everyone, depending on their technical skills. With that in mind, AR might not be suitable for children, the elderly, or the visually impaired. In general, some explanation and practice are needed on how to work the device. Once it was clear how to operate it, it was relatively easy to use.

“*I had to practice a bit. It’s as they say in German: Fingerspitzengefühl*.”

(P1, age 24)

As an explanation is needed before the device can be operated properly, this creates a space at the beginning of the educational session where no substantial information can be given, as students are still trying to master the technique. This can be partially resolved by having the AR models set up beforehand or training students beforehand with the modalities.

##### Didactics

According to students, there are also some didactical aspects that have to be taken into account when implementing AR devices. Group sizes should not be too large: no more than 15 people in total and 3 per device. People need around 20 min to explore three AR modalities, and an instructor should be available to help if questions remain or arise during the experience about the device or the modalities. Furthermore, the space where the AR session is held should be big enough, the lights should be dimmed, and preferably a black background should be used to make the AR model more visible. Finally, there are different ways the AR session can be set up to keep people focused, like using assignments, puzzles, or workstations.

##### Tips and Tricks

When implementing the AR models in either education or the museum, extra explanation on the modalities is necessary. It was not always clear which structure was which, especially because the anatomy of these fetuses deviated from the normal anatomy. Students were able to recognize some structures because of their anatomy knowledge, but they would prefer a more elaborate explanation of the contents of the model. Information could be given by using a color legend, an audio guide, or a popup text. These could be added to the AR devices, and when a certain structure of the model is indicated, information will appear, or an audio fragment will start playing. Furthermore, the AR session should be functional; it should not be a playtime session. This can be difficult as it is a new technique, and students will want to see what the hype is about. Because it is relatively new, there is still room for future developments, however, like being able to see the inside of the organs.

### 3.3. Results of the Analysis for General Visitors

After the themes and categories of the (bio)medical students were defined, the general visitors’ experiences and opinions on the teratological collection and the use of AR were categorized into 18 partially overlapping categories that were sorted into 6 themes (Table 3).

#### 3.3.1. Theme 1: Views on the Teratological Collection

##### Positive Attitude Toward the Collection

The teratological collection was described as beautiful, fascinating, unique, impressive, and interesting to see. It evokes curiosity and stimulates people to think about what is going on with these specimens and what went wrong in their development.

“…*you spend more time looking at a specimen and then it’s mainly interesting and fascinating to see. It makes you think on how it is possible that something can grow like that*.”

(P1, age 24)

##### Negative Feelings and Uneasiness

Negative feelings were mostly present at the beginning of the visit. Once the visitors became used to the collection, these feelings were not as prevalent anymore. The specimens were described as unpleasant, strange, and scary to look at. Some were shocked because they were confronted with real babies. Others even mentioned that they found the specimens monstrous. Visitors pitied the babies and their families; the babies would never have the chance to live and grow up. These feelings caused some uneasiness among visitors.

“*This one with the two heads on one body, it looks a bit monstruous*.”

(P6, age 54)

##### Relevance for the Visitor

General visitors do not always understand the relevance of a teratological collection when there is no additional information available on the specimens. Some visitors do not mind the lack of information, but most stated that when they visit a museum, they do this out of interest in the subject and therefore want to learn about its contents. Without information, visitors cannot gain new knowledge, and that raises the question of whether visiting such a collection is relevant for them. The information that was given on the three specific specimens contributed to the visitor’s understanding of their development. Visitors emphasize the need for information because they cannot always determine what went wrong by just observing the exterior of the specimens.

“…*you see it and interpret it as a simple, technical soul. But you don’t really know what to do with it. I mean you don’t know much about it. And without information you won’t get very far*.”

(P3, age 22)

##### Ethical Aspects

All visitors are glad that the collection is publicly available because when something interests you, you should be able to visit it and possibly learn from it as well. However, they also find it extremely important that it does not become a circus attraction. People should not visit just to observe a “freakshow”; it should be out of interest.

“*If people look at it the right way, then I find it very positive. And educational. But it shouldn’t be a circus attraction. That’s what we had in the past. That was the only way to see them, at the fair*.”

(P4, age 73)

Visitors also realize that parental consent was almost non-existent in the past, but the collection’s educational value is more important than the fact that no consent might have been given. The current ethical standards should not be projected onto something that was conducted in the past, and therefore, visitors stated that it is good that the collection exists and that real examples are being displayed to a broad population.

“*I think that we shouldn’t use the current ethical standards on things that were different in the past. I think we often go too far with that. I think it’s good for people that they can see it in real life*.”

(P10, age 25)

##### Knowledge and Prevention

In general, the visit to the teratological collection was described as educational. The collection is useful to educate both students and general visitors on the development of congenital abnormalities. Exhibiting these specimens also helps create awareness of the existence of such anomalies. Visitors stated that they sometimes did not even know these defects existed before actually seeing them in the museum. These specimens can also serve as a wake-up call for expecting parents. By being confronted with what could happen, this could help them live more healthily during the pregnancy.

“*I want to become a mother too. And when you see this, you realize there are a lot of things that can go wrong*.”

(P10, age 25)

##### Purposes and Use of the Collection

Teratological specimens can be used for different purposes. When used in (bio)medical education, students are required to remember the material, and the given information can be more complex. When general visitors come to a teratological collection, it is not necessarily for in-depth learning; creating enjoyment and awareness are also important factors of their visit. Visitors thus make a clear distinction between educational and museum purposes. Furthermore, specimens can be useful for scientific purposes to investigate how these anomalies developed.

#### 3.3.2. Theme 2: Additional Value of AR

##### Positive Experience with AR

In general, the experience with AR was described as pleasant, fun, and cool. Visitors were impressed with the possibilities of the AR device and with the fact that they were able to see the inside of the specimens. They were also interested in the workings of the device and curious to explore all the options.

##### Learning Efficiency and Insightfulness

The use of AR models is perceived as educational because it provides insights into the inside of the abnormal fetal body. Visitors were able to see what organs were not properly developed and this taught them why the fetuses were not viable, as this was sometimes not clear when only seeing the exterior of the specimens. The use of color also helped the visitor differentiate between the different organs. The AR models thus provided a more in-depth learning environment than the specimens.

“*It is impressive that you can actually see the reason it’s not viable. With the baby with the two heads on one body, you can actually see that it only has one heart*.”

(P12, age 25)

The AR models are seen as dynamic models: structures can be manipulated and viewed from many different angles. This again provides more insights, but it also creates a more interactive way of learning. As visitors were able to decide for themselves which structure “to grab” and explore, they were able to manage their own learning process and create a more personalized learning experience. It urges the visitor to think about the structures and generate a more in-depth understanding of the specimen as a whole.

##### Creating Awareness

The AR devices can serve as an extra factor to create awareness of the existence of different anomalies. It can teach visitors something about the cause of the defects and whether they could have been prevented. It can also be used to inform parents when they are expecting a child with a disability. By showing these disabilities with AR, they might obtain a better understanding of what is wrong with their child.

##### Use of the AR Technique

The technique is generally pleasant and easy to work with after some instructions and practice. There are some drawbacks to user-friendliness that will be discussed in Theme 5. Visitors were impressed with the setup of the AR devices and the corresponding technique. It creates an extra dimension to the learning environment while still being able to see your own surroundings. It is a fun, new technique that visitors interested in AR and teratology can explore. Another advantage is that these models can be (dis)assembled multiple times, unlike the real specimens.

#### 3.3.3. Theme 3: Use of AR in a Teratological Museum

##### Museum Purposes of AR

Almost all general visitors would like to see the AR devices added to the museum visit. Using the AR modalities was enlightening because it provided them with more information. They also mentioned that an interactive museum is more fun and that interactivity helps them remember the things they learned during their visit. It could also help attract a new crowd to the museum. A younger crowd that is interested in AR might mainly see this as an opportunity. Furthermore, it is important to monitor the fact that visitors treat the AR devices and modalities with respect. Visitors also clearly stated that museum purposes are different from educational purposes. People come to a museum to enjoy themselves and acquire some basic knowledge of, for example, teratology. The information for visitors should therefore not be too complex.

“*A museum is mainly for enjoyment and conveying information. Education means further development*…”

(P1, age 24)

##### Ethical Aspects

Ethically speaking, visitors do not mind the AR modalities as much, mostly because they are not that realistic looking. They also mentioned that because they ethically accept the exhibition of human specimens, they also accept these AR models. One visitor mentioned that because the specimens were mostly exhibited without consent, converting them into AR models was ethically not acceptable. It remains important to create ethical awareness when using the AR models to make visitors realize they are still looking at a reflection of a real baby.

“*It looks a bit artificial. That’s why I think the combination, AR as an addition would be good. Because people need to remain aware of the ethical dilemmas and that these are human beings*.”

(P6, age 54)

##### Impact on the Museum Visit

Visitors agree with the students on the subject of using AR modalities to create a more positive museum experience for visitors who perceive the specimens as scary. By using AR first, the initial shock effect can be subdued. Visitors can then form an understanding of the development of such anomalies. When that understanding is present, they might be interested in seeing the real specimen. The AR modalities could serve as an alternative for sensitive visitors, but in general, visitors think it is important for them to see the real specimens as well. If needed, they can be told what to expect by putting a disclaimer at the entrance of the teratological collection.

“*I think it is a way to get rid of your fear. Because you’re always scared of things you don’t know. So, if you know more about it, it becomes less scary*…”

(P12, age 25)

#### 3.3.4. Theme 4: Use of AR in Teratology Education

##### Educational Purposes of AR

Visitors stated that AR can be an added value to the (bio)medical curriculum; it is a new and interesting technology to use. They find it important that students are presented with and use different forms of education. AR models can help students visualize all the structures, and it is also more interactive. When giving them assignments or puzzles while using the AR device, this could stimulate them to learn more about teratology. As long as they do not treat the device as a toy, it can be a good addition. However, when it is used in student education, visitors stated that the AR modalities need further development and should be more detailed, as students are supposed to go more in-depth in their learning than general visitors. Lastly, it was mentioned that besides using AR to teach teratology, it could also be used to practice different operations.

“*Education means further development… I think in education you can go more into depth. And this shouldn’t be the intention of the museum without offering that in-depth information in the medical curriculum*.”

(P1, age 24)

##### AR as an Alternative or Addition to Specimens

Some visitors stated that for students, the AR modalities could serve as alternatives to specimens or a visit to the dissecting room. Students know that these specimens exist, so they are not necessarily needed for teratology education. Also, if a university does not have certain specimens on hand or does not have a teratological museum, AR modalities could serve as a good alternative. Other visitors mentioned that AR should only be used additionally. They still find it important for students to observe the real specimens first to broaden their learning experience and to emphasize awareness. The majority of the visitors found the AR modalities less realistic than the specimens, and this step away from reality could affect how students interact with the learning material.

#### 3.3.5. Theme 5: Improvements and Recommendations on AR

##### User-Friendliness of the AR Devices

There are several aspects regarding AR devices that can be improved. Not everyone can operate such a device and perform the actions associated with it: elderly or non-technical people could experience some trouble. It was also mentioned that a more comprehensive explanation on how to use the device was needed, e.g., how to zoom in or “grab” a structure. Most visitors indicated, however, that after they had some time to practice, they developed the skills to use the device properly. Furthermore, visitors wearing glasses stated that the AR device on top of their own glasses was not always comfortable, and one spectacle wearer developed a headache. The first step in future developments of the AR device should be making it more comfortable and easier to work with.

“*It’s like learning a game and after 15 min you know how to play it*.”

(P4, age 73)

##### Information on the AR Models

Because the specimens and their modalities can deviate quite a bit from the normal fetal anatomy, it was difficult for visitors to recognize certain structures in the AR model, mainly because they do not have the same knowledge as (bio)medical students. One visitor suggested that it could be helpful to project the anatomy of a normal fetus next to the abnormal model. All visitors mentioned that because of the difficulty of the anatomy, they required a substantial amount of information to understand which structures were (ab)normal. If the purpose is to teach visitors about teratology, information on the anatomy of the modalities is a must. This can be performed by creating a color legend; however, colors should then be used in the same way across all structures. Other ways of providing information in the AR device are pop-up texts, audio fragments, and a photo or video displaying the (ab)normal anatomy of the fetuses. Some visitors preferred a guide to be constantly present to provide the information.

“*When you’re using it, it’s interactive, but you don’t know what structures… What you are grabbing. I don’t know. There are probably people that do know. People who are medically versed*.”

(P5, age 53)

##### Technical Developments

AR is a technique that constantly needs to be updated. More details and information can always be added to the AR device. It is mainly the software that should improve; there are delays in the system, the system’s menu is not always clear, and sometimes the system crashes. Furthermore, visitors mentioned that the image of the model is cropped, and they cannot see the entire model when they look downward. Lastly, the system should be properly protected to make sure the data cannot be stolen. If these elements can be resolved, that would be a step in the right direction.

#### 3.3.6. Theme 6: Logistics When Implementing AR

Visitors, like students, mentioned there are logistical factors that have to be considered when implementing AR modalities. The space needs to be big enough for groups, and a dark background is needed to optimize the visibility of the model. When using it in the museum, a separate space should be created where a few devices can be placed for public use. Lastly, cost is the most important factor. For education, most say the cost is worth it because students may be more careful with the devices, but in a museum where everyone can use it, the benefits might not outweigh the costs.

“*I think glasses like this need to be renewed every once in a while. So, you will have to look at the financial picture. If it gets outdated, costs come into play*.”

(P8, age 56)

## 4. Discussion

This is the first qualitative study that describes different aspects that were encountered when museal teratological specimens meet novel innovative methods, such as AR, in detail. We show that exhibiting teratological specimens within a museal setting and the use of AR models are relevant for both (bio)medical students and general visitors for a multitude of reasons. The use of AR models enables visitors to instantly and easily gain deeper knowledge about teratologic specimens.

Anatomy and pathology museums are generally seen as expensive facilities without a well-defined role in medical education [31]. However, both students and general visitors have stated that visiting a teratological collection creates awareness of the existence of these anomalies because they offer real examples. That reality can simply not be achieved with (3D) digital images [18], although this study shows the potential of AR in providing these insights for each type of visitor. It was pointed out that more detailed information on the specimens is desired, especially for visitors without medical expertise. The more uncertain someone is about their knowledge, like general visitors on anatomy, the greater the need for extra information [32]. General visitors do not need as much in-depth information as students, mostly because they visit the museum for enjoyment, but without extra information on the specimens, the museum cannot reach its full potential to educate both students and visitors on teratology. In conclusion, all participants stated that the existence of teratological collections is extremely relevant for a variety of reasons. The use of the AR devices in the museum was perceived as a positive addition by both students and visitors. Previously, AR systems have mostly been tested and used by (bio)medical students, as these devices are sometimes used in anatomy education [33,34,35,36]. One article described the use of AR in a medical museum tour to explore different cross-sections of specimens and enhance motivation for anatomy learning, but again, the device was only used by medical students [22]. In this study, both students and general visitors used AR and described it as a fun and interactive way to gain insights into teratology.

When AR is used in an educational environment, it is known to improve motivation and interest [37]. Students and visitors were more motivated to learn because they were able to create their own learning process. One article suggested that AR systems can also be used for collaborative learning [35]. When used in a group, AR can improve motivation and anatomy learning in general. In our study, all participants used AR individually, but suggestions were made on how it can be used in a group setting. This could be an innovative and dynamic learning method with tremendous learning potential. When working in a group, however, some students could feel like others are watching them while they are manipulating a model that cannot be observed by others. This can generate feelings of awkwardness. A negative feeling like this can cause the student to lose interest in the topic and therefore can interfere with their learning potential [37].

Digital resources, like AR, are mostly seen as a supplement to body dissections or specimens [35,38,39]. In this study, it was also stated that the modalities should mostly be used as an addition because they provide better insights after seeing the specimens. They are also not as realistic-looking as the real specimens. For this reason, the AR modalities could serve as an alternative in case visitors found the real specimens too frightening. The modalities can therefore have a positive impact on the museum visit of these people and create a more visitor-friendly environment. Another possibility is creating more understanding of the topic by using the AR device first. Sensitive visitors can then slowly become accustomed to the subject, after which the visit ends with seeing the real specimens.

It is clear, however, that the AR modalities need to be equipped with additional information. General visitors, even more than students, experienced a lack of information on the anatomy of the modalities. This again can be linked to general visitors having less anatomy knowledge than (bio)medical students, which then leads to a greater need for information [32]. Providing information is a requirement for both museal and educational purposes as both are aimed at educating students and general visitors on teratology. Options like pop-up texts, color legends, and audio fragments were suggested, which could aid the information requirements of all visitors.

The present study also highlights that visiting the collection was always perceived as interesting and educational, even though some participants described the specimens as monstrous and scary. The collection is also useful in the development of healthcare providers to gain knowledge on certain anomalies and to be able to convey this information to others. Nevertheless, several ethical considerations were expressed by students and visitors. Furthermore, it is very rare for museums to explicitly state their ethical reasoning behind exhibiting specimens, even when no consent was given [6]. Our museum in Nijmegen, however, provides some background information on “the when and how” these specimens were collected and how that differs from modern times [6]. The availability of this ethical background is deemed very important, and it should be prominently displayed somewhere in the museum.

One drawback when implementing AR modalities is that users are not trained in the practice of AR devices, and therefore, it can be time-consuming to use [37,40]. Despite this, the AR HoloLens is described as a user-friendly device. Users need some instruction and practice but quickly become acquainted with the technique. Neb et al. (2021) described the fact that even people with disabilities were able to fully grasp the workings of the HoloLens [41]. There were, however, some concerns about the age of the AR users in the museum. Older people could experience more difficulty and therefore would need more guidance [40]. Younger people are usually quicker to become familiar with the functionality of the AR device [41]. However, with the proper instructions, AR could be an appropriate device for everyone. Finally, logistical factors play an important part in whether AR can be successfully integrated in educational settings and a teratological collection. Cost is the main problem. The question remains whether the benefits outweigh the costs. Software problems are also a worry among participants. But as this is a relatively new technology, there are new updates and developments every day that can hopefully resolve these issues. From a modern point of view, it makes sense for medical museums to explore the boundary between old teratological specimens and new AR methodologies in order to use their collections with their optimal educational potential, which is future proof for the next generations to come.

This study is the first to present the views, opinions, and experiences of both (bio)medical students and general visitors regarding a teratological collection and using AR in such a museal setting. The insights of this study can be used by other medical faculties to create a more visitor-friendly museum, to further improve teratological AR modalities, and to implement them in a teratological collection or educational setting. The results of this study form a basis for future quantitative studies on the effect of AR modalities on the understanding of developmental principles that can be encountered in teratological specimens. For future research, a direct comparison with alternatives such as touchscreen-based 3D content could be further explored. Another strength of this study is the in-depth information that was collected from a relatively wide range of visitors. Part of the participating visitors consisted of (bio)medical students from the Radboud University Nijmegen. Their opinions and experiences may not be representative of visitors nationwide. By also including general visitors, this increased the generalizability of the results.

One limitation of this study was that participants only had 20 min to explore the teratological AR modalities. In this relatively short amount of time, they might not be able to fully grasp the concept and use of AR in a museal setting. However, even with limited exposure time to AR, this study could still reveal important insights into the opinions of visitors that could be used in the development of AR models and their integration into a teratology collection and teratology education. In terms of participant selection, because participants volunteered to participate, those visitors might already have a prior interest in AR, teratology, or the human body in itself. Therefore, they might be more enthusiastic to participate, which might lead to a possible source of bias in this study.

### Teratological Specimens: An Ethical Issue for Museums?

Although many European museums display teratological specimens to the general public they rarely clarify the ethical reasoning behind their decision to do so. Most of these specimens were acquired during a time when ethical and moral standards were markedly different from those we uphold today, particularly concerning the treatment of deceased children’s bodies and the necessity of obtaining consent. The diligence to collect these specimens in the past often led to practices that dehumanized the bodies and possibly ignored the wishes of their families [42]. During that era, there were no willful body donation programs, and the norms and values guiding the collection of such specimens differed greatly from contemporary standards. The doctor–patient relationship was typically hierarchical and paternalistic; it was neither expected for physicians to seek parental permission nor common for parents to assert such a right. Concepts like individual autonomy and decisional self-determination were virtually nonexistent [43]. At the time, it was generally considered appropriate to remove a deceased child from the parents’ view as quickly as possible. Most teratological specimens in anatomical museums today were acquired and anonymized in ways that would now be deemed ethically unacceptable [16,17]. Whether or not parents were informed, or gave consent, for the donation of their deceased child remains largely unclear. What information was provided to parents regarding their stillborn fetus is also largely unknown. Historically, such deceased children were often perceived as inert, impersonal bodies. In contrast, contemporary perspectives increasingly recognize and depict them as personified individuals [14]. Although the origins of many teratological collections may seem ethically troubling from a modern standpoint, museums cannot retroactively correct these historical injustices by simply disposing of their collections or making them inaccessible for visitors. These injustices are inherently embedded in the history of every such collection [44]. According to the International Council of Museums (ICOM) Code of Ethics, human remains are considered “culturally sensitive materials.” This designation necessitates thorough ethical reflection and respectful handling at all stages of museum practice—from acquisition to conservation, preparation, and display [45]. Teratological collections are uniquely significant in that they reflect the perceptions, attitudes, and superstitions of their time [16]. While many museums lack precise knowledge of when, how, or why particular specimens were collected, it remains essential to communicate general ethical considerations, contextualized within their historical framework, when deciding to exhibit these materials. Conversely, one might argue that concealing such collections from public view is itself ethically questionable. These specimens, meticulously collected and curated in the past, should not remain hidden, accessible only to a select group of clinicians or researchers. Public exhibition, coupled with transparent ethical discourse, can foster understanding and empathy for the values and practices of earlier times. Moreover, teratological specimens challenge viewers by confronting them with the imperfections of nature and the fragility of life. They also offer a powerful sensory experience through visual and material engagement, which is in itself a very valuable learning experience. While we use our teratological collection in both (public) and (post) academic education, it is a fact that it can be philosophically argued where the threshold lies for determining what education looks like versus morbid curiosity. Evidence of this topical issue lies with the recently published American Association for Anatomy (AAA) Recommendations for the Management of Legacy Anatomical Collections [11] and the increasing number of conference symposia and publications on the topic. While Europe still holds a different opinion around medical collections, ethical issues around the legacy of anatomical collections are becoming more broadly illuminated within the global anatomy community. Finally, it is oftentimes assumed that nothing was asked of parents in the past concerning the donation of their child (personal observations of the first author). However, we do own the consent of parents in approximately 50% of our teratological specimens. As our collection was mainly collected between the 1960s and 1990s it reflects a turning point in the discussion about donation and informed consent.

## 5. Conclusions

This qualitative study indicates that a teratological collection is relevant for a multitude of reasons that mainly focus on providing information and creating awareness on congenital anomalies for both (bio)medical visitors and general visitors. For this reason, teratological collections should continue to exist and be used for educational purposes for both students and general visitors. Furthermore, both (bio)medical students and general visitors are interested in using AR in an educational and museal setting. It was suggested that AR can provide better insights into teratology and create a more interactive way of learning. The opinions and experiences presented in this study can contribute to improving the visitor experience when visiting a teratological collection and can help define the role of AR in such a collection. Ultimately, the exhibition of these specimens holds educational value and may enhance public awareness and acceptance of developmental anomalies, and it provides a unique opportunity to reflect on both historical and contemporary bioethical issues, something which should not be covered up based on modern ethical feelings or thoughts. We feel that AR offers unique affordances, such as spatial anchoring and embodied interaction, which are particularly well-suited to enhancing engagement and learning in physical exhibition spaces such as a teratological collection.

## Figures and Tables

**Table 1:** Participant characteristics.

**Characteristics**	** (Bio)Medical Students **	** General Visitors **
** Gender **		
** -Male (*n*) **	1	4
** -Female (*n*) **	13	8
** Age **		
** -Age range **	20–27 years	22–73 years
** -Median age **	24 years	27.5 years
** Previous experience in the museum **		
** -Present (*n*) **	14	1
** -Not present (*n*) **	0	11
** Previous experience with AR **		
** -Present (*n*) **	3	2
** -Not present (*n*) **	11	10
** Years of (bio)medical education **		
** -1st year (*n*) **	0	/
** -2nd year (*n*) **	3	/
** -3rd year (*n*) **	1	/
** -4th year (*n*) **	2	/
** -5th year (*n*) **	2	/
** -6th year (*n*) **	6	/
** Occupational status **		
** -Working (*n*) **	/	5
** -Non-working (unable, etc.) (*n*) **	/	2
** -Student from non-(bio)medical faculty (*n*) **	/	3
** -Retired (*n*) **	/	2

**Table 2:** Themes and categories for (bio)medical students.

Themes	Categories
Views on the teratological collection.	Additional value of the collection
	Feelings of unease and inconvenience
	Ethical aspects
	Knowledge and prevention
Positive and negative aspects of AR	Positive aspects: positive experience, interactive and insightful
	Negative aspects: logistics and feeling watched
The use of AR in a teratological collection	Impact on museum visit
	AR as an alternative or addition to specimens
Recommendations on using AR devices in museum tours and teratology education	User-friendliness of the AR device
	Didactics
	Tips and tricks

**Table 3:** Themes and their categories for general visitors.

Themes	Categories
Views on the teratological collection	Positive attitude toward the collection
	Negative feelings and uneasiness
	Relevance for the visitor
	Ethical aspects
	Knowledge and prevention
	Purposes and use of the collection
Additional value of AR	Positive experience with AR
	Learning efficiency and insightfulness
	Creating awareness
	Use of AR technique
Use of AR in a teratological museum	Museum purposes of AR
	Ethical aspects
	Impact on museum visit
Use of AR in teratology education	Educational purposes of AR
	AR as an alternative of addition to specimens
Improvements and recommendations on AR	User-friendliness of the AR devices
	Information on the AR models
	Technical developments
Logistics when implementing AR	/

## Data Availability

The original contributions presented in this study are included in the article/Supplementary Material. Further inquiries can be directed to the corresponding author(s).

## Supplementary Materials

The following supporting information can be downloaded at: https://www.mdpi.com/article/10.3390/s25123683/s1, Topic list.
