# Supplementary material for: When Teratology and Augmented Reality Entwine: A Qualitative Phenomenological Analysis in a Museal Setting
Source: Sensors (Basel). 2025 Jun 12;25(12):3683. doi: 10.3390/s25123683 (PMC12197222; doi:10.3390/s25123683)
Supplement: Supplementary file 1 [file sensors-25-03683-s001.zip › sensors-3649434-supplementary.pdf]

sensors

MDPI

Topic guide

General/demographics

How old are you?
What gender do you identify with?
In which academic year are you currently studying (for (bio)medical student visitors)
What is your occupational status at the moment (for non-(bio)medical visitors)
Have you visited the museum for Anatomy and Pathology in Nijmegen before?
Have you previously worked with augmented reality?

Experiences and opinions on teratological collection

How did you experience your current visit to the teratological collection?
What is your opinion on exhibiting teratological specimens in a museum?
Which ethical aspects/ideas come to mind when seeing the teratological specimens?
You received a text from me about the ethical background of the collection. What did you think of that?
What is your opinion on using teratological specimens to help better understand and study congenital anomalies?
What is your opinion on using teratological specimens to create awareness on the existence of congenital anomalies?

Experiences and opinions on the use of augmented reality models in a teratological museum

How did you experience the use of the augmented reality glasses in general?
How did you experience the use of the AR glasses to view the AR teratological models?
How did you experience the extra explanation and commentary you received during the use of the AR glasses and while viewing the AR teratological models?
Which ethical aspects/ideas come to mind when seeing the AR teratological models?
What is your opinion on using AR teratological models in an anatomical museum?
What is your opinion on using AR teratological models to create a more positive museum experience for visitors?
What is your opinion on the use of AR teratological models to create more engagement and interaction for the visitor in the museum?

Opinions on using AR models in teratology education

What is your opinion on using augmented reality models in teratology education?
In your opinion, what are positive aspects of implementing AR in teratology education?
In your opinion, what are negative aspects of implementing AR In teratology education?
What is your opinion on the use of AR to create more engagement and interaction in teratology education?

Additional experiences and opinions

Do you have any additional aspects or questions regarding your museum visit today and the use of AR that you would like to discuss?
